# AI-Driven Prediction of Possible Mild Cognitive Impairment Using the Oculo-Cognitive Addition Test (OCAT) †

**DOI:** 10.3390/brainsci16010070

**Published:** 2026-01-03

**Authors:** Gaurav N. Pradhan, Sarah E. Kingsbury, Michael J. Cevette, Jan Stepanek, Richard J. Caselli

**Affiliations:** 1 Aerospace Medicine and Vestibular Research Laboratory, Mayo Clinic, Scottsdale, AZ 85259, USA; kingsbury.sarah@mayo.edu (S.E.K.); mcevette@mayo.edu (M.J.C.); stepanek.jan@mayo.edu (J.S.); 2 Department of Otolaryngology-Head and Neck Surgery, Division of Audiology, Mayo Clinic, Scottsdale, AZ 85259, USA; 3 Department of Biomedical Informatics, Mayo Clinic Arizona, Scottsdale, AZ 85259, USA; 4 Aerospace Medicine Program, Department of Internal Medicine, Mayo Clinic, Scottsdale, AZ 85259, USA; 5 Department of Neurology, Mayo Clinic, Scottsdale, AZ 85259, USA; caselli.richard@mayo.edu

**Keywords:** mild cognitive impairment, cognitive decline, Dementia Rating Scale, oculometrics, machine learning, feature selection, supervised models, screening

## Abstract

**Background/Objectives**: Mild cognitive impairment (MCI) affects multiple functional and cognitive domains, rendering it challenging to diagnose. Brief mental status exams are insensitive while detailed neuropsychological testing is time-consuming and presents accessibility issues. By contrast, the Oculo-Cognitive Addition Test (OCAT) is a rapid, objective tool that measures oculometric features during mental addition tasks under one minute. This study aims to develop artificial intelligence (AI)-derived predictive models using OCAT eye movement and time-based features for the early detection of those at risk for MCI, requiring more thorough assessment. **Methods**: The OCAT with integrated eye tracking was completed by 250 patients at the Mayo Clinic Arizona Department of Neurology. Raw gaze data analysis yielded time-related and eye movement features. Random Forest and univariate decision trees were the feature selection methods used to identify predictors of Dementia Rating Scale (DRS) outcomes. Logistic regression (LR) and K-nearest neighbors (KNN) supervised models were trained to classify PMCI using three feature sets: time-only, eye-only, and combined. **Results**: LR models achieved the highest performance using the combined time and eye movement features, with an accuracy of 0.97, recall of 0.91, and an AUPRC of 0.95. The eye-only and time-only LR models also performed well (accuracy = 0.93), though with slightly lower F1-scores (0.87 and 0.86, respectively). Overall, models leveraging both time and eye movement features consistently outperformed those using individual feature sets. **Conclusions**: Machine learning models trained on OCAT-derived features can reliably predict DRS outcomes (PASS/FAIL), offering a promising approach for early MCI identification. With further refinement, OCAT has the potential to serve as a practical and scalable cognitive screening tool, suitable for use in clinics, at the bedside, or in remote and resource-limited settings.

## 1. Introduction

Mild Cognitive Impairment (MCI) is a condition that represents a transitional state between normal aging processes and dementia, commonly Alzheimer’s Disease (AD) [1,2,3], though some MCI cases stem from reversible causes like concussion, metabolic pathologies, and psychiatric disorders [4]. Individuals with MCI exhibit cognitive deficits while maintaining functional independence in activities of daily living, as defined by the 2018 American Academy of Neurology guidelines [4]. Diagnostic criteria for MCI initially emphasized memory loss [3], but have evolved to include impairments in other domains, like language, visuospatial processing, or executive skills [2,5], reflecting the heterogeneity of its clinical presentation. Existing cognitive screening tools, such as the Mini-Mental State Examination (MMSE) [6] and the Montreal Cognitive Assessment (MoCA) [7], are commonly employed for MCI detection; however, these tools have notable limitations, including ceiling effects, limited sensitivity to subtle domain-specific deficits, and reliance on language, which may reduce their ability to detect early or non-amnestic forms of MCI. In addition, MMSE and MoCA typically require 10–15 min to administer and must be conducted by trained, certified personnel which introduces additional practical limitations in time-constrained or large-scale screening settings.

Although comprehensive neuropsychological assessment remains the gold standard for MCI diagnosis [8,9], its length, clinical resource requirements, and insurance constraints limit accessibility for many patients. The Mattis Dementia Rating Scale (DRS) is a validated alternative that is frequently used to measure general cognitive function [9,10]; however, administration may require as few as 15–20 min with an alert, healthy individual, but can take close to an hour for a person with advanced impairment [9,11].

There is a growing clinical need for brief, objective digital screening tools to predict a patient’s relative risk for MCI ensuring that those at greatest need are prioritized for further assessment. Changes in eye movements, also termed oculometrics, have been identified as early biomarkers of Alzheimer’s disease, related dementias, and many other neurological diseases, reflecting disruptions in distributed neural networks underlying attention, executive control, and visuospatial processing [12,13]. In response, several digital cognitive assessments have incorporated eye tracking or passive visual paradigms to improve scalability and objectivity. For example, Neurotrack’s Visual Paired Comparison (VPC) task assesses visual recognition memory using eye-tracking and typically requires approximately 30 min to administer [14]. Similarly, CognICA employs brief visual-categorization tasks completed in about 5 min through passive viewing [15]. While these approaches offer efficiency, they are largely constrained to single cognitive domain and rely predominantly on passive engagement, limiting their sensitivity to broader executive and working memory processes that are often affected early in cognitive decline.

The Oculo-Cognitive Addition Test (OCAT) [16], developed by Mayo Clinic researchers, was designed to address these limitations by combining active cognitive engagement with high-resolution oculometric measurement in a single, time-efficient paradigm. Unlike passive viewing tasks, OCAT requires participants to perform verbally administered mental addition tasks under systematically varied cognitive workloads (low, medium, and high), while eye movements are recorded across horizontal, vertical, and diagonal saccadic planes. This design enables simultaneous assessment of multiple cognitive domains including attention, processing speed, executive function, visuospatial processing, working memory, numerical representation, and oculometric coordination all within one minute of testing [17,18]. By integrating cognitive challenge with real-time eye movement tracking, OCAT captures dynamic brain–behavior interactions that are not accessible through single-domain or passive digital assessments.

Importantly, OCAT derives objective, quantifiable oculometric features directly from task performance, minimizing reliance on subjective scoring, examiner expertise, language proficiency, or prolonged administration. In our previous work, we have shown that cognitive impairment can create quantifiable changes in oculometric patterns such as fixation duration, saccadic velocity, blinks, and pupillary response under varying cognitive workloads [17,18,19]. In a proof of concept study, OCAT demonstrated increased saccadic latency and fixation time in a hypoxic population [18], supporting its sensitivity to cognitive load. Together, these findings position OCAT as a scalable, multimodal digital screening tool that complements existing approaches by offering a broader and more mechanistically grounded assessment of cognitive function. A detailed description of OCAT task design and functioning is described in Appendix A [18].

Eye movements, specifically saccades, fixations, blinks, and pupil dilations, have a variety of functions with multiple voluntary and reflexive factors [20,21]. Saccades are rapid, conjugate eye movements that can occur in response to voluntary focus [22] on an object of interest (e.g., during tracking [20,23] or reading [24]) or involuntary effects of cognitive processing (e.g., microsaccades during mental arithmetic [25,26]). OCAT tracks and assesses both voluntary and involuntary saccadic movements. Saccadic latency is the time elapsed from the appearance of a visual target to the initiation of saccadic movement and subsequent visual fixation on the target [20]. Saccadic latencies are increased in patients with Alzheimer’s Disease compared to age-matched control subjects, and latencies also increase with aging [20,27,28,29,30]. Fixation is the ability of a person to sustain visual focus on a target [31]. Abnormalities in fixation (i.e., presence of intrusive saccades during a fixation task) have long been reported as biomarkers of Alzheimer’s Disease [28]. Even though participants are instructed to fixate on the number on the screen during OCAT to ensure data fidelity, involuntary gaze shifting during tasks with high cognitive load is common. Fixation time on a visual target gives insight into the amount of cognitive effort applied on a task [32]. Individuals with suspected MCI also exhibit higher blink rate and increased pupil dilation diameter when completing activities that tax cognitive faculties more heavily [33,34,35]. In our earlier short conference paper, OCAT-derived eye movement features demonstrated predictive ability for the early identification of MCI [36].

While eye-tracking provides valuable oculometrics that serve as sensitive indicators for cognitive assessment, it requires specialized hardware, calibration, and controlled testing environments that may not always be feasible in remote or limited-resource settings. Time-based features—such as total test time, the average time to complete each three-number addition task (where each number is associated with the low, medium, and high cognitive task), and response time of following the number on the screen can be derived independently of eye-tracking hardware and still reflect cognitive processes like attention, processing speed, and working memory. Evaluating the predictive utility of OCAT’s time-based features alone allows for the development of a simplified, accessible version of the test. If effective, this approach could facilitate broader deployment of OCAT as a low-cost, rapid screening tool for cognitive impairment in settings lacking eye-tracking infrastructure.

The objective of this work was to develop predictive machine learning models for possible mild cognitive impairment (PMCI) identification using the time-based and eye movement features extracted from the raw gaze data during the OCAT. OCAT was performed the same day as, but prior to, neuropsychological testing which included the DRS. It is hypothesized that with the optimal model, the selected eye movement and time-related features derived from OCAT will reliably and accurately predict DRS outcomes (PASS/FAIL). Additionally, the development of time-only models is a first step towards cognitive screening without eye-tracking technology, rendering OCAT more generalizable for diverse settings. This study represents the foundational work toward establishing OCAT as a rapid, objective screening tool for neurological function, with the potential to guide further neuropsychological assessment.

## 2. Materials and Methods

### 2.1. Participants

250 participants who underwent neuropsychological testing under the ongoing longitudinal study of the Arizona APOE Cohort [37] at the Department of Neurology in Mayo Clinic Arizona were enrolled in this study protocol, which was approved by the Mayo Clinic Institutional Review Board (IRB). Subjects between 21 and 99 years of age with normal vision (including those with correction), no clinically significant visual impairment, and the ability to provide consent themselves were enrolled. Informed consent was obtained from all participants before enrollment in accordance with Mayo Clinic’s IRB regulations. All tested participants were categorized into Cognitive Normal (CN) and Possible Mild Cognitive Impairment (PMCI) groups, including those with mild dementia, based on their DRS score during clinical neuropsychological assessment. As per the clinical standards, participants with a DRS score of 140 or above were labeled as CN, and those with less than 140 scores were labeled as PMCI [38]. A portion of the data was used to train and optimize supervised machine learning models, while remaining data was reserved for independent validation. Model performance was evaluated by assessing sensitivity, specificity, precision, and accuracy in classifying patients with DRS scores indicative of MCI, based on established diagnostic thresholds [38]. Participants whose eye-tracking data during the OCAT did not meet the predefined “tracking ratio” threshold of 80% due to excessive signal loss, poor eye-tracking calibration, or unstable gaze recordings were excluded from further analysis to ensure the reliability and validity of gaze-based cognitive performance measurements during the OCAT. The final distributed dataset included the OCAT data of 206 participants, with 166 categorized as CN class and 40 as PMCI class. A demographic and clinical characteristics table comparing the CN and PMCI groups is presented in Table 1, summarizing age, sex, education level, and baseline DRS scores.

### 2.2. Equipment

The OCAT was conducted in a quiet, climate-controlled room at Mayo Clinic Arizona. The ambient light levels were at stable luminance and consistent throughout the data collection. The OCAT software (version 1.0) was installed on the 14″ EyeOn Elite Windows 10 Pro tablet (EyeTech Digital Systems, Tempe, Arizona, USA) with an integrated eye tracking device (8MP Eye Gaze Camera) to track eye gaze during the OCAT. The resolution of the tablet screen was 1920 × 1080. The raw eye-gaze data was collected at a sampling rate of 120 Hz. During the study, participants were seated facing the tablet and positioned to maintain a viewing distance of 60 cm, a distance within the recommended reading range. The session began with a 5-point calibration to optimize eye-tracking accuracy. Calibration quality was verified, and recalibration was performed if necessary to maintain a calibration error below 0.5 degrees. To control potential task novelty effects, a structured preview and practice phase were incorporated before data acquisition. Participants first received standardized instructions and observed a demonstration of the task. Subsequently, an initial practice session was conducted, during which participants completed a full OCAT trial that allowed for task familiarization and reduction in learning-related variability. The subsequent OCAT trial was considered the formal test, and only data from this test were analyzed. The OCAT session was performed before any neuropsychological testing schedule for the Neurology appointment to avoid any fatigue effects.

### 2.3. Data Processing and Feature Extraction

The time-related features described in Table 2 were measured during the OCAT for overall task completion time and for each addition sequence. Addition sequences had varying cognitive workloads (low, medium, and high in the form of first, second, and third number, respectively). The raw gaze data obtained during the OCAT were pre-processed prior to extracting eye movement-related features. Pre-processing included artifact removal using filtering and interpolation-based spike correction based on neighboring valid samples to eliminate extreme or physiologically implausible gaze samples. This was followed by applying fixation and saccade classification algorithms based on dispersion thresholds [17]. From the processed data streams, the features related to saccades and fixations, along with blinks and pupillary dynamics were computed to characterize participants’ cognitive and oculomotor performance (Table 2). The features listed in Table 2 were computed for each OCAT performed by every participant. The detailed explanation of eye movement features during OCAT is provided in Appendix B. The features exhibiting significant deviation from a normal distribution (skewness outside the range of −1 to 1) were subjected to logarithmic transformation during the data preprocessing stage. This transformation aimed to reduce skewness and stabilize variance, thereby improving the performance of the classification model and predictive accuracy. It is worth noting that, in addition to the 31 features listed in Table 2, the age of the participants was also included as an additional feature in the predictive modeling.

To develop and evaluate the performance of the machine learning models to predict the possible mild cognitive impairment, the OCAT dataset was randomly split into training and testing sets, with 80% of the data used for training and the remaining 20% reserved for testing. This stratified split ensured that the class distribution between CN and PMCI was preserved across both subsets, as summarized in Table 3. To address the issue of class imbalance in the training data, the Synthetic Minority Over-sampling Technique (SMOTE) was applied. SMOTE generates synthetic samples for the minority class (in this case, PMCI class) by interpolating between existing minority class instances, thereby improving the model’s ability to learn discriminative patterns and reducing bias toward the majority class. Table 4 shows the class distribution of training and testing data after applying SMOTE.

Models were developed and evaluated using both the original imbalanced dataset and a class-balanced dataset generated through SMOTE. Feature combinations included: (1) both time-related and eye movement-related features, (2) time-related features alone, and (3) eye movement-related features alone, to investigate the individual and joint predictive contributions of these feature subsets. Finally, the training data were standardized using the StandardScaler method to ensure that features had zero mean and unit variance. The fitted scaler parameters were saved and subsequently applied to the testing dataset to maintain consistency in feature scaling during model evaluation.

### 2.4. Feature Selection

Feature selection was conducted in a two-step process to reduce redundancy and retain features with high predictive value. First, highly correlated feature groups were identified using Pearson’s correlation coefficient, with a threshold of 0.8, consistent with established practices for multicollinearity reduction [39]. Each group consisted of two or more features that exhibited mutual correlation above this threshold. Within each group, a Random Forest classifier was employed to assess the relative importance of features, and only the feature with the highest predictive importance was retained while the others were discarded. In the second step, a univariate evaluation of the remaining, uncorrelated features was performed using a Decision Tree classifier. Each feature was individually assessed based on its Receiver Operating Characteristic-Area Under the Curve (ROC-AUC) score. Features with ROC-AUC scores less than or equal to 0.5 were excluded, as they contributed no better than random performance in classification.

### 2.5. Learning Models and Analysis Procedure

To predict the likelihood of possible mild cognitive impairment (PMCI) in participants undergoing the OCAT, two supervised machine learning algorithms were employed: Logistic Regression (LR) and K-Nearest Neighbors (KNN). Logistic Regression with L2 regularization and the L-BFGS solver was chosen for its interpretability and effectiveness in binary classification problems, modeling the probability of PMCI occurrence as a function of the selected input features. No class weighting was applied, and default regularization parameters were used. The K-Nearest Neighbors algorithm, a non-parametric method, was used as a complementary approach due to its ability to capture complex, nonlinear relationships in the feature space. Models were implemented using a Euclidean distance metric with uniform neighbor weighting. For LR, the classification threshold was treated as a tunable hyperparameter, and model performance was assessed at two decision thresholds (DT): 0.45 and 0.50, to evaluate the trade-off between sensitivity and specificity. These thresholds were selected a priori to reflect clinically relevant screening scenarios, where prioritizing sensitivity for early risk detection may be desirable, while maintaining reasonable specificity, and were applied consistently across all evaluations, without optimization on the test set. For KNN, the number of nearest neighbors (k) was optimized by evaluating model performance across a range of 3 to 10 values using grid search performed on the training dataset only. The held-out test set was reserved exclusively for final model evaluation.

**Figure 1:**
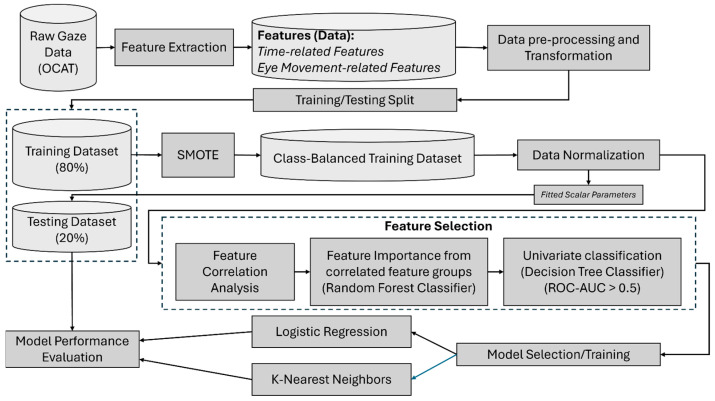
Workflow of the predictive modeling process.

Both models were trained using the preprocessed and feature-selected dataset. The mean values of key performance metrics, including recall, precision, specificity, F1-score, accuracy, and the area under the precision-recall curve (AUPRC) were estimated using bootstrapping with 1000 iterations, with corresponding 95% confidence intervals to characterize stability and uncertainty of predictive performance. Figure 1 illustrates the complete workflow of the model comprising feature extraction, data pre-processing, feature selection, and predictive modeling steps. Pre-processing of the raw gaze data and extraction of eye movement-related features were performed using MATLAB R2024a (Mathworks, Natick, MA, USA). Subsequent feature pre-processing, feature selection, and predictive modeling were implemented in Python (version 3.12.7) using the scikit-learn library (version 1.6.1).

## 3. Results

This study aimed to predict whether a participant would score below 140 on the DRS using the results derived from OCAT, reflecting a higher risk for the possibility of mild cognitive impairment. The classification performance of Logistic Regression (LR) and K-Nearest Neighbors (KNN) was evaluated under both imbalanced (original) and class-balanced (SMOTE-augmented) datasets with different feature combinations described in Table 2.

### 3.1. Results of Prediction Models Using Time and Eye Movement-Related Features

Table 5 shows that the LR model trained on SMOTE-balanced data containing both time-related and eye movement-related features demonstrated superior and consistent performance across all metrics, achieving precision (0.88 [0.75–1]), specificity (0.96 [0.9–1]), and F1-score (0.88 [0.76–0.95]), with high sensitivity (0.87 [0.73–0.91]) and accuracy (0.94 [0.88–0.98]). In contrast, the LR model trained on the original imbalanced data showed reduced recall (0.73 [0.64–0.9] at DT = 0.45 and 0.71 [0.64–0.9] at DT = 0.5), while maintaining high specificity and precision (≥0.88), suggesting a bias toward the majority cognitive normal class.

KNN also benefited from SMOTE, with the balanced model (k = 6) yielding improved recall (0.85 [0.73–1]) and F1-score (0.82 [0.73–0.92]), along with high specificity (0.92 [0.84–1]) and AUPRC (0.9 [0.82–0.98]). Interestingly, the KNN model (k = 5) with original, imbalanced dataset achieved high precision (0.96 [0.75–1]) and specificity (0.99 [0.9–1]), but at the cost of lower recall (0.71 [0.54–0.9]), indicating under-identification of the minority possibly MCI class despite overall high accuracy (0.9 [0.77–0.98]).

**Figure 2:**
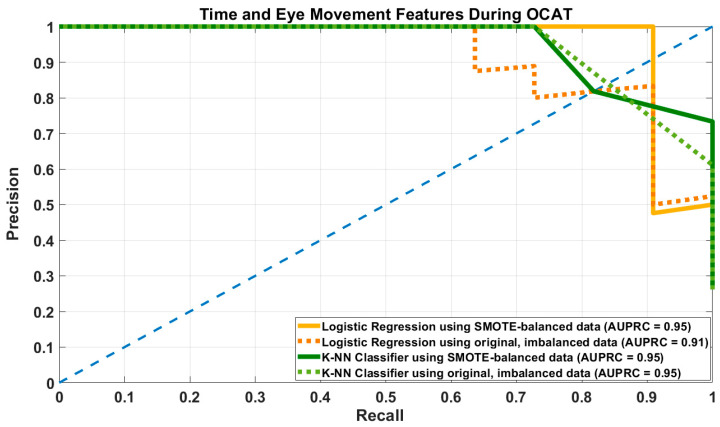
Precision-Recall Curve of Logistic Regression (LR) and K-Nearest Neighbors (KNN) models using both SMOTE-augmented balanced and original, imbalanced datasets with time and eye-movement features during OCAT.

Both models show optimal performance under the SMOTE-balanced condition, as also seen in the precision-recall curve (Figure 2).

### 3.2. Results of Prediction Models Using Eye Movement-Related Features

Table 6 shows the corresponding performance metrics for LR and KNN models with eye movement-related features alone. LR with SMOTE and eye movement features alone achieved the same high recall, but precision, specificity and F1 score were reduced as compared to the models using combined time and eye movement features. Of note, the LR model with imbalanced data using only eye movement features showed higher recall than the combined feature model for both DTs, but precision was decreased, suggesting increased false positives.

**Figure 3:**
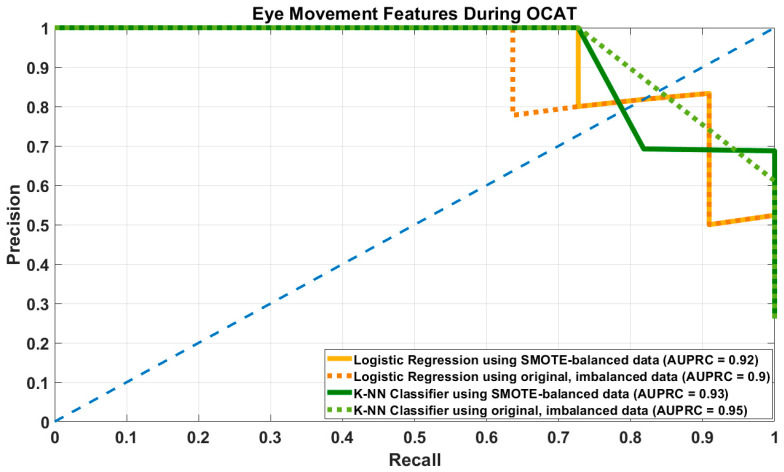
Precision-Recall Curve of Logistic Regression (LR) and K-Nearest Neighbors (KNN) models using both SMOTE-augmented balanced and original, imbalanced datasets with eye-movement features alone during OCAT.

KNN with SMOTE and eye movement features yielded high recall. However, this came at the cost of substantial loss in precision and F1-score, indicating more false positives. The combined feature set offered a more balanced performance, even if recall was slightly lower. When using original data, performance was identical for KNN with either feature set. Figure 3 shows the precision and recall curves for LR and KNN models with SMOTE-balanced and imbalanced datasets with eye movement features alone.

### 3.3. Results of Prediction Models Using Time-Related Features

Table 7 shows the corresponding performance metrics for LR and KNN models with time-related features alone. LR with SMOTE and time-only features retained high precision and specificity but with slightly lower recall than the combined time and eye movement feature set, implying the model was highly conservative and likely under-predicted PMCI cases. This resulted in missed positive cases (false negatives), which could be critical in a clinical setting.

**Figure 4:**
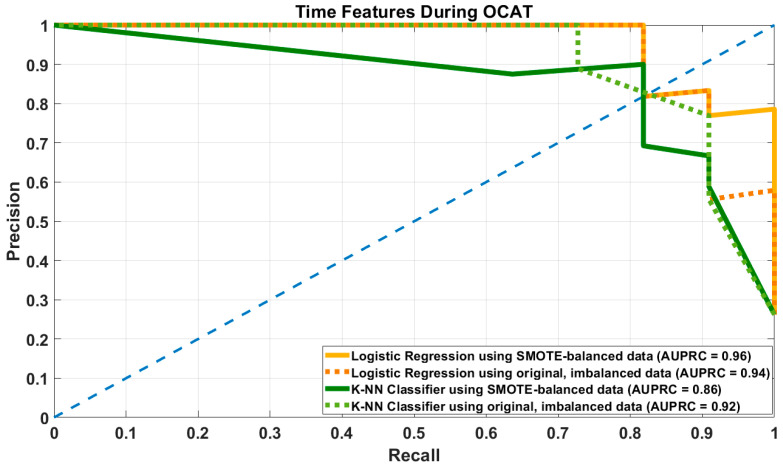
Precision-Recall Curve of Logistic Regression (LR) and K-Nearest Neighbors (KNN) models using both SMOTE-augmented balanced and original, imbalanced datasets with time-related features alone during OCAT.

KNN with SMOTE under time-only features yielded moderate recall and high specificity, but lower precision (0.78 [0.61–0.91]), indicating a noticeable rate of false positives. Both time-only and eye-movement only feature sets performed similarly under KNN, though eye-movement only had better recall. Figure 4 shows the precision and recall curves for LR and KNN models with SMOTE-balanced and imbalanced datasets with time-related features alone.

## 4. Discussion

Multiple predictive models were developed using three OCAT feature groups: eye movement-related features, time-related features, and a combination of both features. Across all groups, logistic regression (LR) models trained on SMOTE-balanced datasets consistently demonstrated the most balanced and robust performance across all metrics. These results highlight the utility of SMOTE in improving recall while maintaining favorable balance between sensitivity and specificity. Notably, the model that incorporated both sets of metrics (eye-movement- and time-related) yielded the best recall, precision, specificity, F1-score, accuracy, and AUPRC of the three groups, indicating that integrating both temporal and oculomotor data offers the most reliable predictive power. Although formal overfitting diagnostics such as learning curves or nested cross-validation were not performed, the relatively narrow 95% confidence intervals obtained via bootstrapping indicated stable internal performance. However, model development and evaluation were conducted within a single cohort, and the absence of external validation limits generalizability. Accordingly, these findings should be interpreted as preliminary, and future studies will focus on validating OCAT-based models in independent cohorts with greater demographic, clinical diversity, longitudinal outcomes to assess reliability, scalability, and clinical utility. In addition, age was included as a feature in the predictive models to account for its known association with cognitive decline [30]. However, feature selection analyses indicated that age did not rank among the most predictive variables relative to OCAT-derived time and eye movement features, suggesting that model performance was not dominated by age effects. These findings support the contribution of OCAT-specific features beyond demographic information, although residual confounding by age cannot be fully excluded and will be further examined in future studies.

In the context of MCI screening, false positives may lead to unnecessary patient expense and stress, as well as provider time, but the screener result does not definitively make the diagnosis. Rather, it rather prompts further evaluation via comprehensive neuropsychological assessment. False negatives, on the other hand, pose a more serious concern in cognitive screening, since missing the indicators of MCI when it is present could delay timely intervention. The DRS is a well-established neurocognitive assessment that can be administered as a standalone measure and comprises multiple subscales spanning attention, initiation/perseveration, construction, conceptualization, and memory [10,40]. It has demonstrated utility in differential diagnosis across dementia subtypes and has been validated as a screening tool for MCI [9,41]. In this study, DRS was used pragmatically as a reference outcome to evaluate the ability of objectively derived OCAT features to predict MCI; however, we acknowledge that the DRS was not designed to serve as a definitive diagnostic standard for MCI and that its use may introduce limitations, particularly in cases of early MCI. Accordingly, future work will evaluate the predictive performance of OCAT against multiple cognitive assessments, including the MMSE, Auditory Verbal Learning Test (AVLT), and other domain-specific neuropsychological measures, as well as clinically adjudicated diagnoses. Additionally, one limitation of this study was the use of a relatively high DRS cutoff (<140) to define the PASS/FAIL outcome. Although this threshold is supported by prior literature [38,42,43,44] as indicative of increased cognitive risk, it likely captures earlier or subtler impairment rather than definitive MCI and may limit direct comparability with studies using lower, more traditional cutoffs (e.g., 123) [9]. This choice was driven by the distribution of DRS scores in the study cohort (Table 1), in which few participants scored in the lower range, but the findings should therefore be interpreted in the context of early cognitive risk rather than established impairment.

These findings support the potential utility of OCAT with integrated eye-tracking as a screening tool for early cognitive impairment, as combining eye-movement and time-based features captures a broader range of cognitively relevant signals. Earlier identification may enable more efficient allocation of clinical resources and timely intervention, particularly in settings where traditional tools lack sensitivity [12,13]. Beyond neurodegenerative conditions, OCAT may also be applicable to other disorders associated with oculomotor dysfunction, such as sports-related concussion, in which saccadic eye movements, accommodation, smooth pursuit (tracking), fixation, and light sensitivity are common [45]. Continued refinement of LR and KNN models may further enhance sensitivity, supporting use in both clinical and sideline settings. This need is especially relevant given the emergence of disease-modifying treatments for Alzheimer’s disease and other causes of cognitive impairment, such as concussions, hypoxia, vascular or metabolic conditions [19,46]. In such cases, early identification and intervention may facilitate cognitive recovery, or prompt additional diagnostic measures, including neuroimaging and further cognitive evaluation [46].

The delays in oculomotor processing that are hallmarks of clinical cognitive decline can also be seen in the central nervous system impairment caused by alcohol, drugs, and fatigue. Alcohol, for instance, produces a central deterioration of nerve impulse transmission that results in systematic impairment of oculomotor functioning [47]. Recent studies have identified measurable eye movement impairments even at sub-intoxicating levels of alcohol, when standard fields sobriety test results are usually negative [48]. Similarly, relationships have been found between the use of cannabis, cocaine, morphine, and phencyclidine to oculomotor impairments, such as increase in saccadic latency and fixations, failure of smooth pursuit, lateral (or vertical) gaze nystagmus, pupil abnormalities and ptosis [49,50]. The current Drug Recognition Expert (DRE) protocol approved by the National Highway Traffic Safety Administration includes an eye-examination step that is highly dependent on the skill of the examiner and lacks objective quantification of the impairment [51]. In this context, a short, minute-long screener like OCAT, especially powered by AI models trained to detect subtle cognitive or oculomotor impairment, could provide a standardized, portable screening tool for use in roadside or other non-clinical settings. Implementing such tools could enhance public safety by supporting more accurate, real-time assessments of functional impairment.

There are valuable practical benefits to evaluating the predictive performance of models based on individual feature groups, especially in the case of time-related features. In SMOTE-balanced datasets, both LR and KNN models using only time-related features achieved moderate recall and high specificity, but the KNN model demonstrated reduced precision, indicating a higher false positive rate. The time-related features include total test time, the average time to complete each three-number addition task (stratified by cognitive load) and response latency inferred from the mean saccadic latency time. These metrics do not require eye tracking hardware and are instead derived from user-initiated clicks to advance to the next number onto the screen. While efforts are increasing to make eye tracking accessible through smartphones [52], the effectiveness of the eye movement features in OCAT remains heavily dependent on clean, artifact-free gaze data. Therefore, the ability of AI models to accurately predict cognitive impairment using only time-related features supports the potential for developing a portable, low-cost OCAT interface suitable for broader clinical, remote, or resource-limited settings. Additionally, this portable application could be deployed in bedside or roadside environments, where eye-tracking best practices might not be able to be practical or consistently maintained [53], broadening OCAT’s accessibility and clinical utility.

## 5. Conclusions

OCAT leverages the well-established relationship between eye movement dynamics and cognitive function to facilitate early detection of cognitive decline associated with MCI, or neurological disorders like Alzheimer’s, other dementias, traumatic brain injuries, substance use, and fatigue. By combining reflexive saccadic eye movements with time-based and attentional effects under varying cognitive load, OCAT provides a multidimensional profile of cognitive performance. As a rapid, non-invasive assessment tool, OCAT can be seamlessly integrated into outpatient clinics, primary care settings, and neurology practices. Its use as an initial screening tool may assist clinicians in identifying patients who would benefit from more extensive evaluations, such as full-extent DRS testing, ultimately conserving time, reducing medical and insurance burdens. With further refinement and dissemination, OCAT could serve as a standardized intake instrument for both preliminary assessment and longitudinal tracking of cognitive state. By featuring OCAT into routine assessments, healthcare providers can enhance early detection, streamline cognitive evaluations, and improve patient outcomes to reduce healthcare costs. The ability to combine time-based and eye movement features enables accurate, rapid screening that may assist neurologists in determining whether more comprehensive neuropsychological testing is warranted. Beyond the clinic, a portable OCAT that captures only time-related features could be used as a roadside examination for drug and alcohol influence testing.

Further development of OCAT will focus on transforming it into a robust AI-powered software platform capable of functioning with or without eye-tracking hardware to enable fast, accurate, and scalable detection of cognitive impairment. Building on the current models, future efforts will explore deep learning approaches to enhance diagnostic accuracy by identifying and classifying the most predictive features, for estimating DRS outcomes and informed care decisions. Continuing validation and model refinement will take place as it is implemented into clinical practice by integrating it into neurological intake appointment workflow at Mayo Clinic, as well as in simulated settings. The OCAT interface will be optimized for usability to be more clinician-friendly and intuitively designed for enabling real-time processing for rapid cognitive assessments. By providing rapid results, OCAT can expedite cognitive assessments in both emergency and clinical settings, reducing the length of neuropsychological evaluations to identify early signs of cognitive decline.

## 6. Patents

Gaurav N. Pradhan, Michael J. Cevette, and Jan Stepanek, Oculo-Cognitive Addition Testing, US Patent 11,869,386 B2, filed 1 December 2016, and issued 9 January 2024.

## Figures and Tables

**Table 1:** Demographic and clinical characteristics of the overall study population and cognitive normal (CN) and possible MCI (PMCI) groups.

	Overall Population *n* = 206	Cognitive Normal (CN) *n* = 166	Possible MCI (PMCI) *n* = 40
Age (years)	65.4 ± 9	64.9 ± 8.9	67.9 ± 9.1
Sex			
Female *n* (%)	144 (70%)	122 (73.5%)	22 (55%)
Male *n* (%)	62 (30%)	44 (26.5%)	18 (45%)
Education years	16.2 ± 2.3	16.3 ± 2.3	16 ± 2.4
DRS	140.9 ± 3.1	142.1 ± 1.5	135.9 ± 3.2

**Table 2:** Description of OCAT features categorized into time and eye-movement related features.

Time-Related Features	Eye Movement-Related Features
Total Test Time (s) Mean Time for Number 1 (low cognitive workload) (s) Mean Time for Number 2 (medium cognitive workload) (s) Mean Time for Number 3 (high cognitive workload) (s) (Log-) Mean Latency Time (ms) (Log-) Standard Deviation of Latency Time (ms)	**Fixations:** Mean Fixation Time for Number 1 (ms) (Log-) Standard Deviation of Fixation Time for Number 1 (ms) Mean Fixation Size for Number 1 (mm) (Log-) Standard Deviation of Fixation Size for Number 1 (mm) Mean Fixation Area for Number 1 (mm^2^) (Log-) Standard Deviation of Fixation Area for Number 1 (mm^2^) Mean Fixation Time for Number 2 (ms) (Log-) Standard Deviation of Fixation Time for Number 2 (ms) Mean Fixation Size for Number 2 (mm) (Log-) Standard Deviation of Fixation Size for Number 2 (mm) Mean Fixation Area for Number 2 (mm^2^) (Log-) Standard Deviation of Fixation Area for Number 2 (mm^2^) Mean Fixation Time for Number 3 (ms) (Log-) Standard Deviation of Fixation Time for Number 3 (ms) Mean Fixation Size for Number 3 (mm) (Log-) Standard Deviation of Fixation Size for Number 3 (mm) Mean Fixation Area for Number 3 (mm^2^) (Log-) Standard Deviation of Fixation Area for Number 3 (mm^2^)
	**Saccades:** 19. Median Diagonal Saccadic Velocity (deg/s) 20. Median Horizontal Saccadic Velocity (deg/s) 21. Median Vertical Saccadic Velocity (deg/s)
	**Blinks:** 22. Blink Rate (number of blinks per minute) 23. Median Blink Duration (ms)
	**Pupillary Dynamics:** 24. Standard Deviation of Pupil Size (mm) 25. Coefficient of Variation of Pupil Size

**Table 3:** Class distribution of training and testing dataset from the original, imbalanced dataset.

Classification	Cognitive Normal (CN) Class	Possible MCI (PMCI) Class
Training dataset	135	29
Testing dataset	31	11

**Table 4:** Class distribution of training and testing dataset after applying SMOTE to get an augmented, class-balanced dataset.

Classification	Cognitive Normal (CN) Class	Possible MCI (PMCI) Class
Training dataset	135	135
Testing dataset	31	11

**Table 5:** LR and KNN mean model performance with bootstrapped 95% confidence intervals on SMOTE-balanced and imbalanced datasets for combined time and eye movement features.

Model	Hyper- Parameter	Recall/ Sensitivity	Precision	Specificity	F1-Score	Accuracy	AUPRC
LR–SMOTE	DT = 0.45	0.88 [0.73–0.9]	0.87 [0.71–1]	0.95 [0.87–1]	0.87 [0.76–0.95]	0.93 [0.88–0.98]	0.94 [0.9–0.96]
	DT = 0.5	0.87 [0.73–0.91]	0.88 [0.75–1]	0.96 [0.9–1]	0.88 [0.76–0.95]	0.94 [0.88–0.98]	
LR–Original	DT = 0.45	0.73 [0.64–0.9]	0.88 [0.7–1]	0.89 [0.87–1]	0.8 [0.7–0.9]	0.9 [0.85–0.95]	0.91 [0.84–0.96]
	DT = 0.5	0.71 [0.64–0.9]	0.91 [0.73–1]	0.97 [0.9–1]	0.79 [0.7–0.9]	0.91 [0.86–0.95]	
KNN–SMOTE	Best k = 6	0.85 [0.73–1]	0.8 [0.66–1]	0.92 [0.84–1]	0.82 [0.73–0.92]	0.9 [0.86–0.95]	0.9 [0.82–0.98]
KNN–Original	Best k = 5	0.71 [0.54–0.9]	0.96 [0.75–1]	0.99 [0.9–1]	0.81 [0.63–0.9]	0.91 [0.83–0.95]	0.9 [0.77–0.98]

**Table 6:** LR and KNN mean model performance with bootstrapped 95% confidence intervals on SMOTE-balanced and imbalanced datasets for eye movement features alone.

Model	Hyper- Parameter	Recall/ Sensitivity	Precision	Specificity	F1-Score	Accuracy	AUPRC
LR–SMOTE	DT = 0.45	0.9 [0.81–0.91]	0.77 [0.64–0.91]	0.9 [0.84–0.97]	0.83 [0.72–0.91]	0.9 [0.83–0.95]	0.93 [0.88–0.96]
	DT = 0.5	0.89 [0.81–0.91]	0.79 [0.67–0.91]	0.94 [0.84–0.97]	0.84 [0.86–0.95]	0.91 [0.86–0.95]	
LR–Original	DT = 0.45	0.8 [0.64–0.91]	0.84 [0.73–1]	0.94 [0.9–1]	0.81 [0.7–0.91]	0.9 [0.86–0.95]	0.92 [0.86–0.96]
	DT = 0.5	0.78 [0.64–0.91]	0.85 [0.73–1]	0.95 [0.9–1]	0.81 [0.7–0.91]	0.88 [0.86–0.95]	
KNN–SMOTE	Best k = 6	0.87 [0.73–1]	0.74 [0.64–0.9]	0.89 [0.84–0.97]	0.79 [0.7–0.9]	0.88 [0.83–0.95]	0.88 [0.79–0.97]
KNN–Original	Best k = 5	0.73 [0.54–0.91]	0.97 [0.67–1]	0.98 [0.87–1]	0.83 [0.66–0.95]	0.92 [0.83–0.98]	0.91 [0.8–0.99]

**Table 7:** LR and KNN mean model performance with bootstrapped 95% confidence intervals on SMOTE-balanced and imbalanced datasets for time features alone.

Model	Hyper- Parameter	Recall/ Sensitivity	Precision	Specificity	F1-Score	Accuracy	AUPRC
LR–SMOTE	DT = 0.45	0.83 [0.82–0.91]	0.9 [0.75–1]	0.96 [0.9–1]	0.86 [0.78–0.91]	0.93 [0.88–0.95]	0.96 [0.93–0.98]
	DT = 0.5	0.82 [0.72–0.91]	0.95 [0.81–1]	0.98 [0.94–1]	0.88 [0.82–0.9]	0.94 [0.9–0.95]	
LR–Original	DT = 0.45	0.73 [0.64–0.81]	0.99 [0.89–1]	0.99 [0.98–1]	0.84 [0.78–0.9]	0.93 [0.9–0.95]	0.93 [0.89–0.98]
	DT = 0.5	0.72 [0.64–0.81]	0.99 [0.98–1]	1 [0.98–1]	0.84 [0.78–0.9]	0.93 [0.9–0.95]	
KNN–SMOTE	Best k = 6	0.81 [0.73–0.91]	0.78 [0.61–0.91]	0.91 [0.81–0.97]	0.79 [0.69–0.91]	0.89 [0.81–0.95]	0.86 [0.76–0.94]
KNN–Original	Best k = 5	0.73 [0.54–0.81]	0.89 [0.67–1]	0.96 [0.87–1]	0.8 [0.67–0.9]	0.9 [0.83–0.95]	0.88 [0.76–0.94]

**Figure A1:**
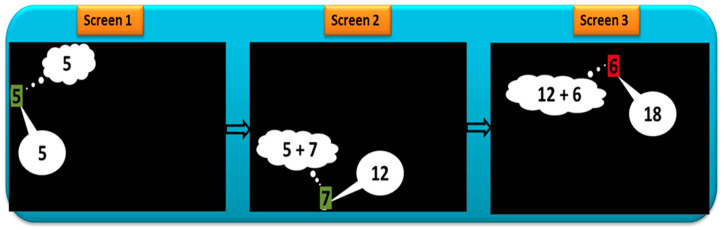
A visual representation of one trial of OCAT. 12 trials of OCAT are completed by each subject with a different set of three numbers in three different locations. Screens 1, 2, and 3 correspond to low, medium, and high cognitive workload respectively. The cloud represents cognitive processing, and the oval represents the verbal response of the subject.

**Figure A2:**
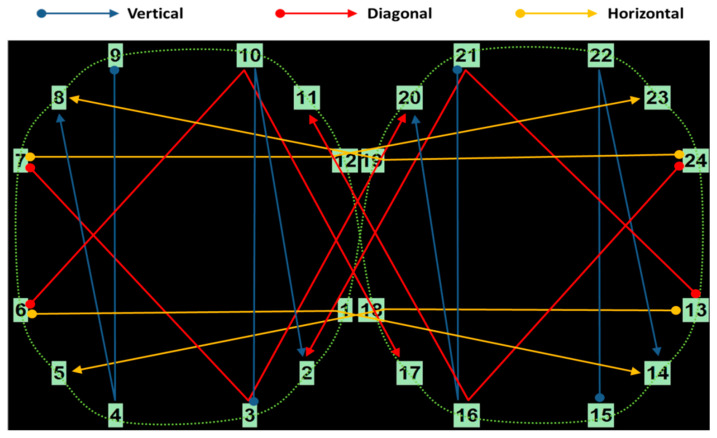
All 12 unique combinations of trials in three categories: vertical (blue), diagonal (red), and horizontal (yellow).

**Figure A3:**
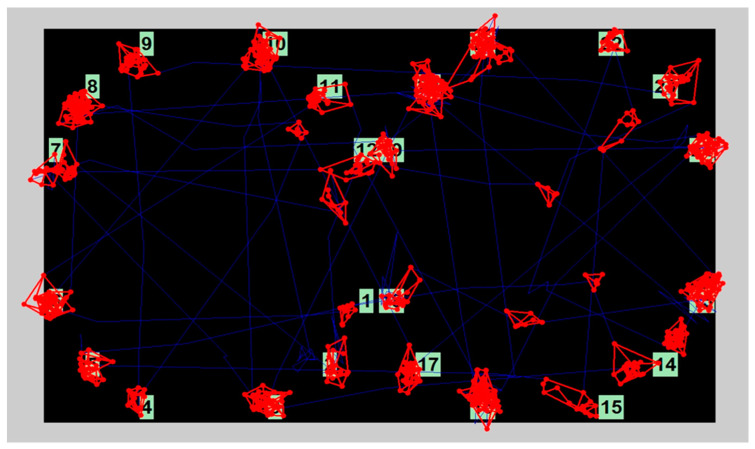
Eye tracking data during OCAT showing the fixations (in red) and saccadic movement (in blue).

## Data Availability

The raw data supporting the conclusions of this article will be made available by the authors on request.

## Abbreviations

The following abbreviations are used in this manuscript:

MCI	Mild cognitive impairment
OCAT	Oculo-Cognitive Addition Test
PMCI	Possible Mild Cognitive Impairment
CN	Cognitive Normal

## Appendix A

### Oculo-Cognitive Addition Test

Oculo-Cognitive Addition Test (OCAT) consists of completing, as rapidly as possible, 12 trials of summing three consecutive numbers shown separately on three consecutive blank screens in seemingly random positions (Figure A1). OCAT tasks the subject with low, medium, and high cognitive workload in each trial using simple mental addition. Low cognitive workload occurs when the subject simply locates, reads, and calls out the number displayed on the first screen (e.g., 5). Medium cognitive workload occurs when the subject locates a second, single digit number and performs mental arithmetic by recalling the first number, adding it to the second number, and calling out the sum of the two numbers on the second screen (e.g., 5 + 7 = 12). Finally, high cognitive workload occurs when the subject locates a third, single digit number on the third screen and is then tasked with the more difficult mental arithmetic task of recalling the sum of the first two numbers, adding it to the third number, and calling out the total sum of all three numbers (e.g., 12 + 6 = 18). Each trial consists of a different set of three numbers at different locations.

To the subject, the position of the number on each screen appears random and hence requires visual scanning since the subject is unable to anticipate the location of the upcoming number. However, while the location of the numbers may appear random to the subject, OCAT consists of structured “infinity loop” pattern of 24 symmetrical positions in which the numbers appear (Figure A2). By utilizing this “infinity loop” pattern, OCAT is inherently structured, allowing us to measure and quantify eye movements in horizontal, vertical, and diagonal directions. Furthermore, since the pattern appears random to the subject, OCAT is also able to overcome the predictability inherent in other cognitive tests that are designed with a linear structure.

## Appendix B

### Appendix B.1. Eye Movement Features

While the subject completes OCAT, an eye tracking device records the raw scan-paths of the subject’s eye movements. Figure A3 demonstrates an example of raw scan-paths. The red clusters represent fixations which are generated when the subject is fixated on the number. The blue lines represent saccadic movements which are generated when the subject locates a new number between screens. Using these raw scan-paths, researcherscan further extract oculometrics related to fixations and saccades. For further details regarding oculometric extraction algorithms, refer. This raw gaze data obtained during the OCAT were pre-processed to extract eye movement-related features. Pre-processing involved cleaning artifacts using filtering techniques followed by applying fixation and saccade classification algorithms based on dispersion thresholds [20] followed by extracting oculometrics related to classified fixations and saccades.

### Appendix B.2. Data Related to Fixations

For each trial of OCAT, we measured average fixation time (ms), average fixation size (mm) and average fixation area (mm^2^) separately during low, medium, and high cognitive workload tasks across all 12 individual trials. The fixation time represents the temporal characteristic of fixations, whereas fixation size and area represent the spatial characteristics of fixations.

The fixation time, also commonly known as fixation duration, is the amount of time that the subject visually fixed on a position. The fixation size is the total spatial scan path length of eye movement within a fixation, in other words, the distance micro-saccadic movements travel within a fixation. The fixation area measures the spatial dispersion of the fixation and is given by calculating the area of the convex hull encompassing all sampled eye-tracking data points within a fixation. For each trial of OCAT, we also calculated the standard deviation of fixation time, size, and area within the 12 trials during low, medium, and high cognitive workload as a measure of fluctuation in fixation time, fluctuation in fixation size, and fluctuation in fixation area, respectively.

### Appendix B.3. Data Related to Saccades

As seen in Figure A1, within each of the 12 trials, three consecutive numbers are presented on a blank screen one at a time such that the user has to perform a saccadic eye movement in either the horizontal, vertical, or diagonal direction to move between each number (i.e., four trials for each of the three directions). For each OCAT, we measured average saccadic velocity (deg/s) in horizontal, vertical, and diagonal directions. We also measured average saccadic latency, which is a metric of the reaction time of fixating from one number to the next. Unlike fixations, measures related to saccades were not stratified according to cognitive load since saccades occur between tasks, presumably when the subject is not tasked with any cognitive load.

The average saccadic velocity is the mean of all saccadic velocities along a given direction during OCAT. And the average saccadic latency is a measure of the time delay between stimulus onset, i.e., number appearing on the screen, and initiation of the subsequent saccade.

### Appendix B.4. Data Related to Blink and Pupillary Dynamics

From the raw eye gaze recordings, several pupil-based and blink-related features were extracted to capture physiological responses potentially indicative of cognitive load. To derive blink-related features from raw eye gaze data, blink events were identified based on interruptions or loss of valid pupil detection, typically indicated by missing or invalid gaze samples over short durations. A blink was defined as a continuous sequence of absent gaze data lasting between 100 ms and 500 ms, aligning with physiologically plausible blink durations. The blink rate was computed as the total number of such detected blink events per minute of OCAT task engagement, providing a measure of blink frequency. For each participant, the duration of individual blinks was calculated, and the median blink duration was extracted to characterize central tendency while minimizing the influence of outlier events. These blink-derived features offer insight into oculomotor behavior and cognitive load during the OCAT task.

The standard deviation of pupil size was calculated from continuous pupil diameter measurements over the course of the OCAT task, reflecting intra-individual variability in pupil dynamics and potential sensitivity to task demands. To quantify cognitive stress responses, the coefficient of variation of pupil size was calculated as the ratio between the standard deviation of the pupil diameter and the mean pupil diameter during OCAT.
